# Personal Neoantigens From Patients With NSCLC Induce Efficient Antitumor Responses

**DOI:** 10.3389/fonc.2021.628456

**Published:** 2021-04-13

**Authors:** Wei Zhang, Qi Yin, Haidong Huang, Jingjing Lu, Hao Qin, Si Chen, Wenjun Zhang, Xiaoping Su, Weihong Sun, Yuchao Dong, Qiang Li

**Affiliations:** ^1^ Department of Pulmonary and Critical Care Medicine, Shanghai Changhai Hospital, Second Military Medical University, Shanghai, China; ^2^ Department of Pulmonary and Critical Care Medicine, Shanghai East Hospital, Tongji University, Shanghai, China; ^3^ Department of Emergency, Shanghai Changhai Hospital, Second Military Medical University, Shanghai, China; ^4^ School of Basic Medicine, Wenzhou Medical University, Wenzhou Tea Mountain Higher Education Park, Wenzhou, China; ^5^ Biotherapy Center, Qingdao Central Hospital, The Second Affiliated Hospital, Qingdao University, Qingdao, China

**Keywords:** neoantigen, neoantigen-reactive T cells (NRTs), non-small cell lung cancer (NSCLC), tumor vaccine, immunotherapy

## Abstract

**Objective:**

To develop a neoantigen-targeted personalized cancer treatment for non-small cell lung cancer (NSCLC), neoantigens were obtained from collected human lung cancer samples, and the utility of neoantigen and neoantigen-reactive T cells (NRTs) was assessed.

**Methods:**

Tumor specimens from three patients with NSCLC were obtained and analyzed by whole-exome sequencing, and neoantigens were predicted accordingly. Dendritic cells and T lymphocytes were isolated, NRTs were elicited and IFN-γ ELISPOT tests were conducted. HLA-A2.1/K^b^ transgenic mice were immunized with peptides from HLA-A*02:01^+^patient with high immunogenicity, and NRTs were subjected to IFN-γ, IL-2 and TNF-α ELISPOT as well as time-resolved fluorescence assay for cytotoxicity assays to verify the immunogenicity *in vitro*. The HLA-A*02:01^+^lung cancer cell line was transfected with minigene and inoculated into the flanks of C57BL/6^nu/nu^ mice and the NRTs induced by the immunogenic polypeptides from autologous HLA-A2.1/K^b^ transgenic mice were adoptively transfused to verify their immunogenicity *in vivo*.

**Results:**

Multiple putative mutation-associated neoantigens with strong affinity for HLA were selected from each patient. Immunogenic neoantigen were identified in all three NSCLC patients, the potency of ACAD8-T105I, BCAR1-G23V and PLCG1-M425L as effective neoantigen to active T cells in suppressing tumor growth was further proven both *in vitro* and *in vivo* using HLA-A2.1/Kb transgenic mice and tumor-bearing mouse models.

**Conclusion:**

Neoantigens with strong immunogenicity can be screened from NSCLC patients through the whole-exome sequencing of patient specimens and machine-learning-based neoantigen predictions. NRTs shown efficient antitumor responses in transgenic mice and tumor-bearing mouse models. Our results indicate that the development of neoantigen-based personalized immunotherapies in NSCLC is possible.

**Precis:**

Neoantigens with strong immunogenicity were screened from NSCLC patients. This research provides evidence suggesting that neoantigen-based therapy might serve as feasible treatment for NSCLC.

## Introduction

Non-small cell lung cancer (NSCLC) is the most common cause of cancer related death worldwide, accounting for more than one million deaths annually. The current standard treatment for NSCLC is oncogene-targeted therapy or debulking surgery combined with paclitaxel and carboplatin chemotherapy. Despite a good initial response, most patients relapse and ultimately develop resistance, and no curative therapeutic options are currently available (1, 2). Immunotherapies that boost the ability of endogenous T cells to destroy cancer cells have demonstrated therapeutic efficacy in a variety of human malignancies (3). Immunotherapy using agents such as immune-checkpoint inhibitors (ICI) has been a focus of attention (4) and their effectiveness in the treatment of NSCLC has been reported (5–10). The emergence of these therapeutic agents has greatly advanced the treatment of lung cancer. However, even for patients with PD-1 ligand (PD-L1) expression ≥50%, only 30% of them can benefit from anti-PD-1 treatment. So, it is very important to explore new immunotherapy methods for NSCLC.

In cancer, mutations are the source of neoantigens that can be recognized by the immune system as foreign-like peptides called ‘neoepitopes’ presented on major histocompatibility complex (MHC) molecules (11). However, the nature of the antigens that allow the immune system to distinguish cancer cells from noncancer cells has long remained elusive. Recent technological innovations have made it possible to dissect the immune response to patient-specific neoantigens that arise as a consequence of tumor-specific mutations, and emerging lines of data suggest that the recognition of such neoantigens is a major factor contributing to the activity of clinical immunotherapies. In melanoma and glioblastoma, the targeting of select neoepitopes by vaccination has demonstrated high immunogenicity and signs of clinical efficacy (12, 13). Although the cancer mutanome is considered a possible source of potent tumor antigens for cancer immunotherapy, it has remained largely out of reach for decades due to the lack of suitable genomic and proteomic methods to identify actionable mutations. Lung cancer genomes harbor somatic mutations that are caused by exposure to mutagens such as smoking (14). The density of somatic mutations and neoantigens was recently shown to correlate with long-term benefits from immune checkpoint blockade in non-small cell lung cancer (NSCLC) (15). However, the study and application of neoantigen-based immunotherapy in NSCLC have been rarely reported (16, 17).

In this study, tumor specimens and blood samples from three NSCLC patients were collected, and whole exome and transcriptome sequencing were conducted. Gene mutations, gene expression, and patient HLA typing were analyzed using various software programs, and the aberrant peptides were prioritized according to the identified mutation, HLA typing and gene expression information. Multiple putative mutation-associated neoantigens with strong affinity for HLA were selected from each patient, and *in vitro* experiments and NRT-induced cytotoxicity *in vivo* evaluation assays were performed. All the patients developed NRT cells responses against multiple vaccine neo-epitopes. Our study demonstrates that individual mutations can be exploited in NSCLC, and this finding opens a path toward personalized immunotherapy for patients with NSCLC.

## Materials and Methods

### Patient Material and Cell Lines

Tumor samples from NSCLC patients were obtained from biopsy specimens. A portion of the sample was removed for formalin fixation and paraffin embedding (FFPE). The remainder of the tissue was immediately frozen in aliquots and stored in vapor-phase liquid nitrogen.

PBMCs obtained for immune monitoring or as starting material for the manufacturing process were isolated by Ficoll-Hypaque (Amersham Biosciences) density gradient centrifugation from buffy coats of healthy donors or from peripheral blood samples of NSCLC patients. Immature dendritic cells (DCs) were generated as described previously (18, 19). T2 and H522 cells (ATCC) were cultured under standard conditions. T2 and H522cells were stably transfected with the A2.1/Kb chimeric gene (T2/Kb, H522/Kb) for mice experiments. T2/Kb and H522/kb cells were stable transfectants and express the product of the HLA-A*02:01/Kb chimeric gene (the α1 and α2 domains from HLA-A*0201 and the α3 domain of H-2K^b^ (20). Cell banks were generated and tested for mycoplasma. The cells were reauthenticated short tandem repeat (STR) profiling at ATCC.

### Next-Generation Sequencing

For WES sequencing, DNA from fresh frozen tumor samples or cells was extracted using the DNeasy Blood and Tissue Kit, which was purchased from Qiagen. The genomic DNA was sheared, end-repaired, ligated to barcoded Illumina sequencing adapters, amplified, and size-selected. Whole-exome capture was performed using an Agilent Sure Select Human All Exon 44-Mb version 2.0 bait set (Agilent Technologies) (21). The resulting libraries were then quantified by qPCR, pooled, and sequenced using 76-basepaired-end reads obtained with HiSeq 2000 or 2500 sequencers (Illumina).

For RNA sequencing, RNA from fresh frozen tumor samples was extracted using the RNeasy Mini Kit. RNA-seq libraries were prepared using an Illumina TruSeq Stranded mRNA Library Prep Kit (for cell suspensions). Flow cell cluster amplification and sequencing were performed according to the manufacturer’s instructions using either a HiSeq2500.

The sequencing data have been deposited in the NCBI Sequence Read Archive (SRA) database under the accession code SRP (https://submit.ncbi.nlm.nih.gov/subs/sra/SUB8559404/overview).

### Bioinformatics and Mutation Discovery

All mutanome-related data analysis steps for a single patient were coordinated by a software pipeline implemented in the Python programming language. At least 150× 10^6^and 75× 10^6^ paired-end 50 nt reads were required for the DNA and RNA libraries, respectively. For mutation detection, the DNA reads were aligned to the reference genome hg19 with BWA (22). Duplicate exomes from tumor and matched normal samples were analyzed for single-nucleotide variants. Loci with putative homozygous genotypes in the normal samples were identified and filtered to retain high-confidence calls for single-nucleotide variants. The remaining sites were further inspected for a putative homozygous or heterozygous mutational event. The suspected sites were filtered to remove potential false positives, and replicates were incorporated by testing both the sum of replicates and the replicates separately. The final list of single-nucleotide variants was composed of high-confidence homozygous sites in the normal samples and high-confidence heterozygous or homozygous mutational events in the tumor samples. The genomic coordinates of identified variants were compared with known gene transcript coordinates detailed in the UCSC Genome Browser to determine the association of the variants with genes, transcripts, potential amino acid sequence changes and RNA-seq-derived expression values.

For RNA-seq, RNA reads were aligned to the hg19 reference genome and transcriptome using Bowtie (23), and the gene expression levels were determined by comparison with known gene transcript and exon coordinates detailed in UCSC followed by normalization to RPKM units (16).

## HLA Typing

The HLA alleles of each patient were inferred from the WES data using OptiType (24) with the default settings after the reads were filtered by aligning to the HLA region using RazerS version 3.4.0 (25).

## Neoantigen Identification

Non-synonymous somatic mutations, including SNVs, insertion, deletion and frameshift, were used to predict neoantigens following the previous procedure with modification (26). First, 8-11-amino-acid long mutant peptides were generated for MHC I-restricted neoantigen prediction, and 12-15-amino-acid long mutant peptides were generated for MHC II-restricted neoantigen prediction. Then HLA binding affinity for each peptide was calculated by NetMHCpan4.0 (27) and NetMHCIIpan3.2 (28) for MHC I and MHC II molecules respectively. Expression level of mutant genes was derived from RNA-seq data in transcripts per million (TPM). The number of mismatches between the mutant and normal peptides was considered as the similarity to self-peptides. Mutant allele frequency was detected by the variant caller MuTect2 (29). Each peptide was given a priority score based on HLA binding affinity, expression level, similarity to self-peptides, the final score for each neoantigen was calculated as follows:

$$\begin{array}{l} \text{NeoantigenScore}\;(\text{p})=\text{affinityMutantScore}\;\text{ExpressionScore}\;\text{VAF}\;\mathrm{Normal Exact Match Penalty}\\ \text{affinity Mutant Score}\;=1/\left(1+\text{math}.\text{exp}\left(5*\left(\text{float}\left(\text{rank}\_\text{mutant}\right)-2\right)\right)\right)\\ \text{ExpressionScore}=\{\begin{array}{l} 1,\;\text{if}\;\text{TPM}>\text{upper}\;\text{quartile}\\ 0.5,\;\text{if}\;\text{lower}\;\text{quartile}\;<\;\text{TPM}<\text{upper}\;\text{quartile}\\ 0.25,\;\text{if}\;\text{TPM}<\text{lower}\;\text{quartile}\\ 0,\;\text{TPM}=0 \end{array}\\ \text{NormalExactMatchPenalty}=\text{Normal}\;\text{exact}\;\text{match}\;\text{penalty}:0\;\text{if}\;\text{mutated}\;\text{peptide}\;\text{matches}\;100\%\;\text{to}\;\text{any}\;\text{peptide}\;\text{in}\;\text{the}\;\text{reference}\;\text{proteome},\;\text{else}\;1. \end{array}$$

Peptides with priority score larger than 0 were selected as neoantigen candidates. Peptides with a %rank less than 0.5 were considered strong binders and peptides with a %rank larger than 0.5 were considered weak binders.

## Synthesis of Long Peptides and Final Vaccine Preparation

Peptides with purity greater than 95% were synthesized by GL Biochem (Shanghai, China) using fluorenyl methoxy carbonyl chemistry by reverse-phase high-performance liquid chromatography and were confirmed by mass spectrometry. The lyophilized peptides were dissolved in dimethyl sulfoxide, diluted in phosphate-buffered saline (pH 7.4) to a concentration of 10 mM and stored as aliquots at −80°C as described previously.

### Minigenes

A tandem minigene (TMG) was constructed as previously described (30). Minigenes were included in each TMG construct used in this study. Plasmids encoding the minigenes were linearized with the restriction enzyme NsiI, and each linearized plasmid was used as a template for *in vitro* transcription using the mMESSAGE mMACHINE T7 Transcription Kit (Thermo Fisher Scientific) according to the manufacturer’s instructions.

### Induction of Neoantigen-Reactive T Cells by Coculture With NSCLC Patient-Derived Peripheral Blood Lymphocytes (PBLs) *In Vitro*

The autologous PBMCs from the patients were harnessed to evaluate the immunogenicity of the candidate neoantigens *in vitro*. An established simple and effective culture protocol with a few modifications (31, 32) was applied to detect and monitor the antigen peptide-specific cytotoxic T lymphocyte precursors (CTL-P) in the circulation. Briefly, heparinized blood samples were obtained from patients with relapsed/refractory tumors for the isolation of PBMCs by centrifugation on a Ficoll density gradient, and the isolated PBMCs were suspended in AIM-V medium (Gibco). In each U-bottomed well of a plate, 1 × 10^5^ PBMCs were incubated with a corresponding peptide (25 μM) in 200μl of culture medium, which was applied to facilitate cell-to-cell contact. The culture medium consisted of AIM-V medium, 10% FCS (Gibco), and IL-2 (100 U/ml; PeproTech). For peptide stimulation, half of the culture medium was replaced with fresh medium containing the corresponding peptide (25 μM) and IL-2 (100 U/ml) at 3-day intervals. After three cycles of peptide stimulation followed by overnight re-stimulation, the specific T cell responses to each peptide were evaluated through an ELISPOT assay on day 10. The recognition of each single antigen was tested in comparison with the no-peptide control (medium only), and phytohemagglutinin was used as the positive control. In some cases, the reactivity of T cells was evaluated by peptide pulsing of DCs cocultured with T cells. Mature DCs were pulsed with 10 μM peptide for 4–6 hours at 37°C, washed with prewarmed PBS, and then incubated overnight with T cells at a stimulator/effector ratio of 1:10 in complete AIM-V medium. IFN-γ-secreting cells was determined by IFN-γ ELISPOT assay.

### Generation of Neoantigen-Specific T Cells in HLA-A2.1/K^b^Tg Mice

The preparation of bone marrow-derived DCs from Tg mice and the vaccination of HLA-A2.1/K^b^Tg mice (10 mice per group)with peptide-pulsed DCs were performed as described previously (33). On day 7 after the last immunization with peptide-pulsed DCs, all the immune splenocytes from the same group of primed mice were collected, cultured at a density of 1 × 10^7^ cells per well in six-well plates and stimulated with peptides (20 μM) for 7 days *in vitro*. The bulk populations were functionally tested by enzyme-linked immunospot assay (ELISPOT), enzyme-linked immunosorbent assay (ELISA), intracellular staining assays, and time-resolved fluorescence assays.

### ELISPOT Assay

ELISPOT kits (Dakewei) was used to determine the amount of cytokine-secreting T cells after overnight activation with a peptide (34). In this study, a multiple culture protocol was used to analyze the T cell response as described above. Briefly, the peptide-stimulated PBMCs or the DC-pulsed peptide coculture with T cells (10^5^ per well) were added to duplicate wells for 18–20 hours. The plates were washed before addition of the diluted detection antibody (1:100 dilution) and then incubated for 1 hour at 37°C. After the plates were washed, streptavidin–HRP (1:100 dilution) was added, and the plates were incubated at 37°C for 1 hour. 3-Amino-9-ethylcarbazole (AEC) solution mix was then added to each well, and the plates were left in the dark for approximately 15–25 minutes at room temperature. Deionized water was then added to stop the reaction, and the plates were subsequently scanned using an ELISPOT CTL Reader (Cellular Technology Inc.). The results were analyzed with ELISPOT software (AID). Spots with a size that were more than twofold greater than that of the no-peptide control (medium only) were considered positive for T cell reactivity.

### *In Vitro* T Cell Cytotoxicity Assay

Cytotoxicity assays were performed using time-resolved fluorescence assay as previously described (35, 36). Briefly, target cells were labeled with a fluorescence enhancing ligand (BADTA)and co-incubated with NRT cells for 2h. The supernatants were then measured by time-resolved fluorometry (EnVision 2014Multilabel reader, PerkinElmer). The percent specific lysis was determined according to the following formula: (experimental release −spontaneous release)/(maximum release −spontaneous release)) × 100% and the percent spontaneous release was calculated from(spontaneous release – background signal)/(maximum release − background signal) × 100%.

### Adoptive Immunotherapy inTumor-bearing Nude Mice

Splenocytes from each group of immunized HLA-A2.1/K^b^ mice were stimulated with 20 μMACAD8-T105I, BCAR1-G23V or PLCG1-M425Lfor 7 days as described in the section detailing the protocol used for the cytotoxicity assay. HLA-A*02:01^+^H522/Kb-minigene tumor cells (5 × 10^6^) were injected s.c. into the flanks of C57BL/6nu/nu mice, which formed homogeneous tumors in 100% of the mice. Three days later (37–41), the mice were intravenously injected with splenocytes (1 × 10^8^ cells per mouse) from each group of immunized HLA-A2.1/K^b^ mice that were stimulated with 20 μM ACAD8-T105I, BCAR1-G23V and PLCG1-M425L for 7 days as described in the section detailing the protocol used for the cytotoxicity assay. This adoptive transfer was performed twice at 1-week intervals and was followed by the intraperitoneal administration of 2000 U of hIL-2 every 2 days. The control mice received splenocytes from HLA-A2.1/K^b^ mice immunized with irrelevant peptide -pulsed DCs or only IL-2. The tumor size and body weight were measured in two perpendicular dimensions three times at weekly intervals. The mice were killed 80 days after tumor inoculation.

### Statistical Analysis

GraphPad Prism 5.0 (GraphPad Software) was used for all statistical analyses. The data samples were compared using two-tailed Student’s t test, and a *P* value less than 0.05 was considered significant.

## Results

### Immunogenic Neoantigens Were Predicted and Identified From NSCLC Patients

**Figure 1:**
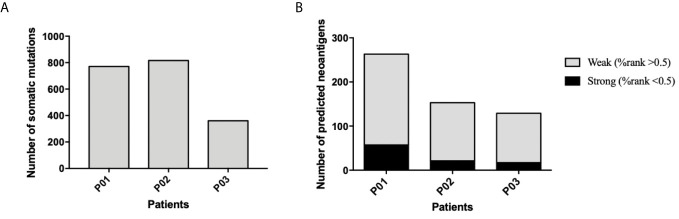
Frequency of somatic mutations and predicted neoantigens in 3NSCLC patients. **(A)** WES and RNA-seq were performed in 3 patients with NSCLC. Tumor-specific non-synonymous somatic mutations were identified. The frequency of somatic mutations of each patient is shown. **(B)** Neoantigen prediction was performed for each patient. The frequency of neoantigens as well as strong binder (%rank <0.5) and weak binder (0.5< %rank <2) of each patient is shown.

Whole-exome sequencing of tumor specimens and matched normal samples from three NSCLC patients (Clinicopathological characteristics of the subjects were presented in **Supplementary Table 3**) was performed after DNA extraction, and the resulting genetic mutations and HLA typing information were then analyzed (**Supplementary Table 1**). RNA was also extracted, and transcriptome sequencing was performed to confirm the genetic mutations and identify mutant gene expression level. Patient somatic mutations and RNA expression were analyzed by MuPeXI to identify neoantigen candidates. An average of 184 non-synonymous somatic mutations were detected (**Figure 1A**) and an average of 145MHC I-restricted neoantigens (range of 97-226) and an average of 38 MHC II-restricted neoantigens (range of 32-46) were identified in 3 NSCLC patients (**Supplementary Table 2**). An average of 34 neoantigens were considered as strong binders with %rank less than 0.5, and an average of 150 neoantigens were considered as weaker binders with %rank larger than 0.5 (**Figure 1B**). Ten putative mutation-associated long peptides (27 aa) with strong HLA affinity were selected from each patient and produced by chemosynthesis (**Table 1**).

**Table 1:** *In silico* prediction of mutations of three NSCLC patients with favorable HLA class I or II binding properties.

Patient	Gene	HLA restriction	Mutated sequence^a^	Substitution (WT, AA#, Mut)	HLA- I or -IIScores^b^
	POLE	B*1502	SRYFHIPIGNLPE**Y**ISTFGSDLFFARH	D1663Y	88
	FHL1	A*0206	CFTCSNCKQVIGT**V**SFFPKGEDFYCVT	G139V	86
	GPM6B	DQA1*0401	TILCFSGVALFCG**F**GHVALAGTVAILE	C94F	81
	LRP5	B*8101	CSHICIAKGDGTP**L**CSCPVHLVLLQNL	R1237L	80
P01	DMXL1	B*1502	QLRENFQEKRQWL**F**KYQSLLRMFLSYC	L2124F	78
	TP53	B*8101	CMGGMNRRPILTI**F**TLEDSSGNLLGRN	I3123F	70
	TPBG	A*0206	SAPFLASAVSAQP**L**LPDQCPALCECSE	P57L	84
	HACE1	DRB1*1101	QLNRLTRSLRRAR**S**VELPEDNETAVYT	T20S	70
	COQ3	A*0206	RYPWARLYSTSQT**A**VDSGEVKTFLALA	T91A	68
	RAB4A	DRB1*1101	ERMGSGIQYGDAA**F**RQLRSPRRAQAPN	L199F	53
	ITFG1	A*0207	CVFILAIIGILHW**L**EKKADDREKRQEA	Q591L	39
	OPLAH	DQA1*0501	EGAVFLSFKLVQG**D**VFQEEAVTEALRA	G890D	49
	BCAR1	DPA1*0202	RQGIVPGNRLKIL**L**VVPTRVGQGYVYE	V64L	31
	TP53	A*3303	TIITLEDSSGNLL**V**RNSFEVRVCACPG	G266V	32
P02	GBF1	B*1501	SSQHASRGGQSDD**Y**EDEGVPASYHTVS	D1478Y	25
	COP1	B*5801	ILWDGFTGQRSKV**S**QEHEKRCWSVDFN	Y485S	31
	ATP11B	DQA1*0301	LKNTKEIFGVAVY**S**GMETKMALNYKSK	T262S	29
	UPF3A	A*3303	GSQDSGAPGEAME**T**LGRAQRCDDSPAP	R380T	26
	IER5L	A*0207	LHKNLLVSYVLRN**T**RQLYLSERYAELY	A43T	30
	SNX16	B*5801	QDVWMRSRADNKP**Y**LSFSEPENAVSEI	C318Y	27
	OSBPL6	A*0201	EVLLSASSSENEA**L**DDESYISDVSDNI	S513L	19
	NFE2L2	A*0201	AFFAQLQLDEETG**Q**FLPIQPAQHIQSE	E82Q	18
	SLX4	A*0201	SPTKEAPPGLNDD**G**QIPASQESVATSV	A1694G	18
	ACAD8	A*0201	QTDVGGSGLSRLD**I**SVIFEALATGCTS	T105I	31
	MTREX	A*0201	EMPKLTEQLAGPL**C**QMQECAKRIAKVS	R933C	15
P03	BCAR1	A*0201	VLLSWKVLDFSGP**V**PQGTGQPCSCGHW	G23V	37
	SLC7A1	A*0201	KYAVAVGSLCALS**S**SLLGSMFPMPRVI	A349S	28
	PLCG1	A*0201	SIEDHCSIAQQRN**L**AQYFKKVLGDTLL	M425L	23
	PIF1	A*0201	EADLFDKLEAVAR**G**VRQQNKPFGGIQL	A325G	3
	SSH1	A*0201	ILDASKQRHNKLW**C**QQTDSSLQQPVDD	R470C	2

NSCLC, nonsmall cell lung cancer. ^a^Mutated residues are highlighted in hold. WT, wide type; AA#, position of mutated amino acid; Mut, mutation. ^b^HLA class I and II binding affinity are predicted by netMHCpan 4.0 and netMHCIIpan 3.2, respectively.

**Figure 2:**
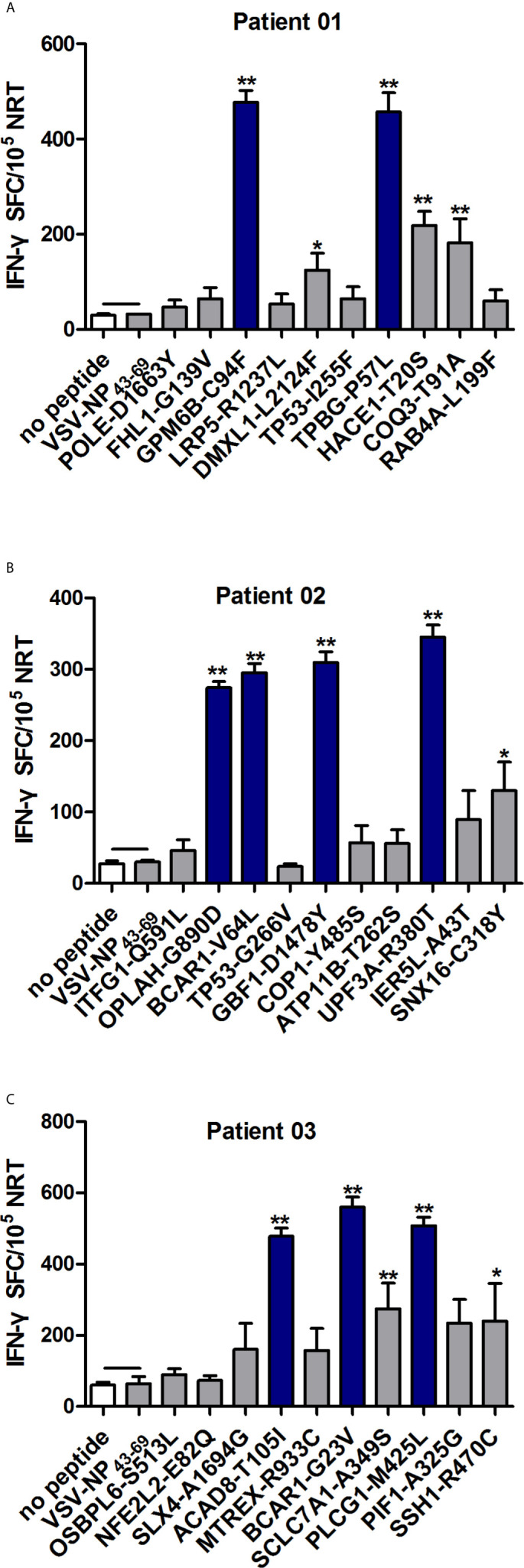
Evaluation of the immunogenicity of neoantigens from patients with NSCLC. Autologous PBMCs were stimulated with candidate mutated peptides every 3 days in the presence of IL-2, and on day 10, the T cell responses to each antigen were measured by IFN-γ ELISPOT assay. The PBMCs in **A–C** were obtained from NSCLC patients 01, 02, and 03, respectively. Stimulation with the no-peptide control (medium only) or irrelevant peptide VSV-NP_43-69_(STKVALNDLRAYVYQGIKSGNPSILHI) was performed as controls. The data are presented as the means ± s.e.m.s from three independent experiments. **P<0.01 and *P<0.05 were obtained for the comparison of IFN-γ production by PBMCs stimulated without a peptide or with irrelevant peptide VSV-NP_43-69_. SFC, spot-forming cell; VSV-NP, vesicular stomatitis virus expressing influenza nucleoprotein.

In order to identify the neoantigen with high immunogenicity, an established simple and effective polypeptide immunogenicity assay with a few modifications (31) was applied to test the immunogenicity of the synthesized neoantigen polypeptides based on NSCLC patient-derived PBMCs. The neoantigens GPM6B-C94F, DMXL1-L2124F,TPBG-P57L,HACE1-T20S and COQ3-T91A from HLA-0206^+^ Patient 01 (P01), OPLAH-G890D, BCAR1-V64L,GBF1-D1478Y,SNX16-C318Y and UPF3A-R380T fromHLA-0207^+^ and HLA-3303^+^ Patient 02(P02) andACAD8-T105I, BCAR1-G23V,SCLC7A1-A349S, SSH1-R470C and PLCG1-M425L from HLA-0201^+^ Patient 03(P03) elicited more obvious peptide-specific T-cell responses than the no-peptide control (medium only) or the irrelevant peptide control (VSV-NP43-69, STKVALNDLRAYVYQGIKSGNPSILHI), as shown by ELISPOT assay (**Figures 2A–C**). GPM6B-C94F and TPBG-P57L from P01, OPLAH-G890D, BCAR1-V64L, GBF1-D1478Y and UPF3A-R380Tfrom P02, and ACAD8-T105I, BCAR1-G23V, and PLCG1-M425Lfrom P03manifestedparticularly distinct peptide-specific T-cell responses.

### Neoantigen-Specific T Responses Can Be Induced *In Vitro* From PBLs of HLA-A*02:01^+^Patient 03 With NSCLC

**Figure 3:**
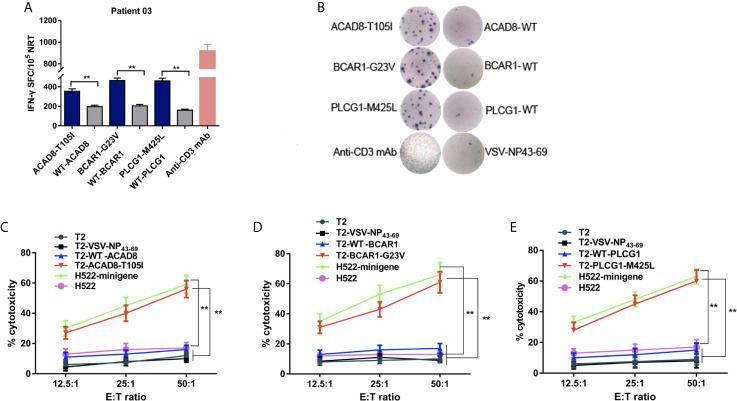
Cytotoxicity of NRTs obtained from the *in vitro* stimulation of PBLs of HLA-A*0201-positive patient 03 with NSCLC. NRTs were induced with autologous ACAD8-T105I, BCAR1-G23V and PLCG1-M425L-pulsed DCs from PBLs of Patient 03 (P03). Seven days after the third stimulation, the NRTs were harvested for analysis. **(A, B)** IFN-γ secretion by neoantigen-reactive T cell lines against mutated and wild-type peptides. IFN-γ-positive SFCs/10^5^ NRTs detected by cytokine-specific ELISPOT assay. Stimulation with anti-CD3-mAb was used as positive control. **(C–E)** Cytotoxicity at the indicated E:T ratios measured using time-resolved fluorescence assay. Mutant peptide-pulsed T2 cells and minigene-nucleofected H522 cells (HLA-A2.1^+^, H522-minigene) were used as peptide-specific targets, whereas irrelevant peptideVSV-NP43-69-pulsed T2 cells,T2 cells alone and H522 cells were used as controls. The data are presented as the means ± s.e.m.s from three independent experiments. **P<0.01. NRTs, neoantigen-reactive T cells; WT, wild-type peptide; E:T, effector: target; PBL, peripheral blood lymphocyte; SFC, spot-forming cell; VSV-NP, vesicular stomatitis virus nucleoprotein.

In order to explore the role of NRT in patients with NSCLC.DCs from P03 (HLA-A*02:01^+^) loaded with ACAD8-T105I, BCAR1-G23V and PLCG1-M425L were cocultured with PBLs to generate mutated peptide-specific NRTs *in vitro*, and the immune responses were compared with the WT peptide-induced NRTs. As shown by ELISPOT assay, the means spots of IFN-γ-producing T cells were significantly greater in NRTs elicited by the aberrant peptides than in those induced by the WT peptides (**Figures 3A, B**).

Mutant peptide-pulsed HLA-A*02:01^+^ T2 cells and minigene-nucleofected H522 cells (HLA-A*02:01^+^, H522-minigene) were used as peptide-specific targets to further examine the cytotoxic effect of the corresponding NRTs. The killing activity of NRTs at different ratios of effective cells to target cells is shown in **Figures 3C–E**. Bulk NRTs against ACAD8-T105I, BCAR1-G23V and PLCG1-M425L, particularly NRTs against BCAR1-G23V and PLCG1- M425L, exerted significant cytotoxic effects toward target cells expressing corresponding antigens. No significant cytotoxic effect was detected with T2 cells that did not pulse any peptides or pulsed irrelevant peptides (VSV-NP43-69) and H522 cells that did not express any mutant peptides.

### HLA-A2.1-Restricted Neoantigens Can Induce NRT Cells in HLA-A2.1/k^b^ Mice *In Vivo*

**Figure 4:**
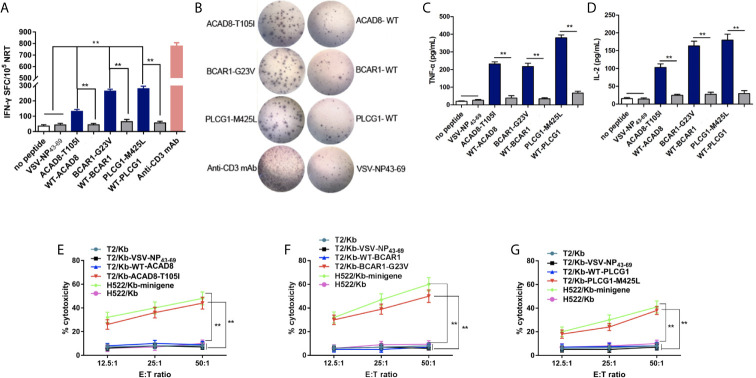
ACAD8-T105I, BCAR1-G23V and PLCG1-M425Linduce more efficient NRT responses than WT epitopes in HLA-A2.1/K^b^Tg mice. **(A–D)** Splenocytes of mice(n=5) vaccinated with mutated peptides were tested by ELISPOT assay for the recognition of mutated peptides compared with the corresponding wild-type sequences. The data are presented as the means ± s.e.m.s from three independent experiments. **P < 0.01 was obtained in the comparison of IFN-γ **(A, B)**, TNF-α **(C)** and IL-2 **(D)** production by splenocytes stimulated without a peptide or with VSV-NP43-69. **(E–G)** Splenocytes from HLA-A2.1/K^b^Tg mice immunized with mutated peptides were re-stimulated *in vitro* with the corresponding mutated peptide for 7 days. The ex vivo cytotoxicity against the corresponding mutated peptide-pulsed T2/Kb cells and minigene-nucleofected H522/Kb cells at the indicated E:T ratio was examined using time-resolved fluorescence assay. Irrelevant peptide VSV-NP43-69-pulsed T2/Kb cells, T2/Kb cells alone or H522/Kb cells were used as controls. Stimulation with anti-CD3-mAb was used as positive control. The data are presented as the means ± s.e.m.s from three independent experiments. **P<0.01. E:T, effector: target; SFC, spot-forming cell; VSV-NP, vesicular stomatitis virus nucleoprotein.

To evaluate the immunogenicity of the candidate polypeptides *in vivo*, the polypeptides ACAD8-T105I, BCAR1-G23V and PLCG1-M425L from P03 (HLA-A*02:01^+^), whose immunogenicity was previously proven *in vitro*, were selected for the immunization of HLA-A2.1/k^b^ mice. On days 0 and 7, ACAD8-T105I, BCAR1-G23V and PLCG1-M425L (100 μg per peptide) were mixed with 50 μg of poly (I:C), and the mixture was subcutaneously injected into transgenic mice for immunization. Splenocytes were collected 7 days after the last immunization. Some of the splenocytes were used for IFN-γ, TNF-αand IL-2 ELISPOT assay. The rest of the splenocytes were cultured for another 7 days with the corresponding peptides, and T cells were then isolated for cytotoxicity detection. NRTs against ACAD8-T105I, BCAR1-G23V and PLCG1-M425L from HLA-A2.1/K^b^ mice contained more IFN-γ, TNF-α and IL-2-producing T cells than those obtained using the WT epitopes, as demonstrated by ELISPOT assay (**Figures 4A–D**). The no-peptide control (culture medium only) and the irrelevant peptide control (VSV-NP43-69) only induced baseline-level secretory responses. In the cytotoxicity assay, the neoantigen-induced NRTs, particularly those induced with BCAR1-G23V and PLCG1-M425L, presented higher cytotoxicity against T2/Kb cells and H522/Kb-minigene cells carrying or expressing the corresponding mutant peptides. No significant cytotoxicity was detected in T2/Kb cells that were not loaded with the peptide or were loaded with irrelevant peptides or H522/Kb cells that did not express mutant peptides (**Figures 4E–G**).

## Adoptive NRT immunotherapy of C57BL/6^nu/nu^ mice bearing human NSCLC

**Figure 5:**
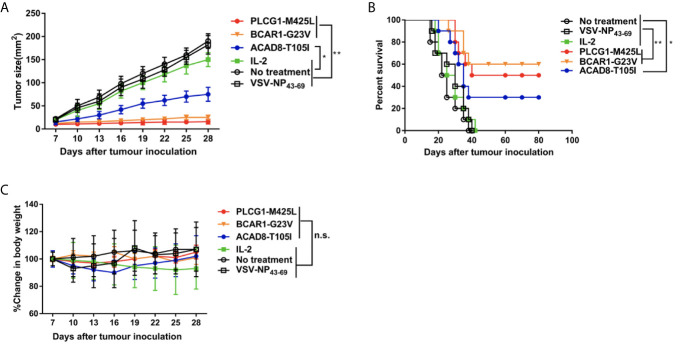
Adoptive immunotherapy of minigene-nucleofectedH522/Kb tumor-bearing nude mice. Minigene-nucleofected H522/Kb tumor cells (5 × 10^6^ cells/mouse) were injected into the flanks of C57BL/6^nu/nu^ mice. Three days later, splenocytes (1×10^8^ cells/mouse) from ACAD8-T105I-, BCAR1-G23V- or PLCG1-M425L-immunized HLA-A2.1/K^b^Tg mice were injected as described in the Materials and methods section. The mice in the control groups received IL-2 alone or did not receive treatment. **(A)** Tumor growth was observed every 3 days and recorded as the mean tumor size (mm 2). **(B)** Survival of mice after tumor inoculation (n = 10 mice/group). **(C)** The effect of different treatments on mouse body weight. (*P<0.05; **P<0.01).

To further investigate whether ACAD8-T105I, BCAR1-G23V and PLCG1-M425L peptides can serve as potent vaccines against tumor growth *in vivo*, HLA-A2.1/K^b^ transgenic mouse-derived NRTs were adoptively transferred into H522/Kb-minigene human lung cancer-bearing C57BL/6 nude mice. As shown in **Figure 5A**, tumor-bearing nude mice treated with neoantigen (ACAD8-T105I, BCAR1-G23V and PLCG1-M425L)-induced NRTs exhibited significantly delayed tumor growth, whereas the irrelevant peptide-induced NRTs did not restrict tumor growth. In addition, 6/10 (60%) of the BCAR1-G23V vaccination group, 5/10 (50%) of the PLCG1-M425L vaccination group and 3/10 (30%) of the ACAD8-T105I vaccination group exhibited prolonged long-term survival (over 80 days) after tumor inoculation (**Figure 5B**), whereas all the mice in the control groups died between days 19 and 40. With regard to the toxicity as detected by the loss of body weight, adoptive NRT immunotherapy seemed to be well tolerated because no appreciable body weight loss were observed in all treatment groups (**Figure 5C**). The above-mentioned findings indicated that the neoantigens ACAD8-T105I, BCAR1-G23V and PLCG1-M425L can potently and safely induce anti-tumor responses *in vivo*.

## Discussion

Tumor-specific somatic mutations are considered ideal targets for tumor immunotherapy, but the study and application of neoantigen-based immunotherapy in NSCLC have not been reported. To develop neoantigen-targeted personalized cancer treatments for NSCLC, samples of human lung cancer were collected, and the potential use of selected neoantigens as targets for NSCLC immunotherapy was tested. In accordance with our research, immunogenic neoantigen were identified in all three NSCLC patients. Additionally, T cells activated by neoantigens from patient 03 (HLA-A*02:01^+^) showed anti-tumor potency, as demonstrated with HLA-A2.1/K^b^ transgenic mice and tumor-bearing mouse models.

With the rapid development of immunotherapy, numerous studies have attempted to develop cancer vaccines (42). Neoantigens are a series of immunogenic peptides derived from tumor-specific mutations or viral open reading frames rather than from the normal human genome (43–45). Neoantigens are highly immunogenic and can escape from central thymic tolerance. Four successful phase I clinical trials were recently and have attracted much attention for use in the development of personalized neoantigen vaccines (12, 13, 46, 47). Although neoantigens represent the optimal choice for anti-tumor immunotherapy, their application is hindered by the unique neoantigen landscape of individualized tumors. The density of somatic mutations and neoantigens was recently shown to correlate with long-term benefits from immune checkpoint blockade in NSCLC (48). Neoantigen loss is observed during immune checkpoint blockade and has implications for the development of immune therapies that target tumor neoantigens (15). Karasaki et al. (14). found a median of 46potential neoantigens (pNeoAgs) (range of 13-659) for adenocarcinoma and 95.5 pNeoAgs(range of 10-145) for squamous cell carcinoma (SCC) in NSCLC. It was previously shown that the selection pressures from a diverse tumor microenvironment affect neoantigen presentation, tumor-intrinsic mechanisms that lead to immune escape and their respective effects on clinical outcomes in NSCLC (49). These studies have confirmed the existence of abundant neoantigens in lung cancer. However, the role of neoantigens in lung cancer remains unclear. Neoantigens were predicted from all three lung cancer patients, and nine from thirty selected neoantigens, which induce significant specific T cell responses with a patient’s own PBMC cells, were also confirmed in our study. Our results shown higher positive ratio than the average level reported by Daniel K. et al. (50), which may be related to the cancer type, ethnic specificity, health/immune condition, and randomization effect (51).

Recently, increasing evidence has shown that adoptive T cell therapy, specific for neoantigens, has been successfully used to treat many human solid cancers (16, 52). Rosenberg led his research team to identify neoantigens in a patient with metastatic cholangiocarcinoma who exhibited radiotherapy and chemotherapy failure using a whole-exome sequencing-based approach (30).Subsequently, the same research team published another paper in NEJM describing the successful application of adoptive TIL cell therapy in a patient with metastatic colorectal (53). In another study, Chen F. et al. (16) successfully treated advanced cancer patients with multiple metastases using personalized peptide vaccines in combination with NRT adoptive therapy, and these results further verified the application of individualized tumor therapy based on NRTs. All these observations verified the potency of putative neoantigens in the induction of NRTs, which also indicated the potential of patient-specific immunotherapy approaches, particularly adoptive T cell transfer, in the treatment of lung cancer. In accordance with our research, *in vitro* experiments and NRT-induced cytotoxicity *in vivo* evaluation assays showed that all the NSCLC patients developed NRT cell responses against multiple vaccine neo-epitopes.

Our research confirms the presence of large amounts of neoantigens in NSCLC. Functional neoantigens were successfully screened through gene sequencing, software prediction and other evaluations. Specific NRTs were successfully elicited, and their cytotoxicity was confirmed both *in vitro* and *in vivo*. Our study provides evidence showing that neoantigen-based therapy might serve as feasible treatment for NSCLC that show great potential for achieving maximal therapeutic specificity, overcoming immune tolerance, and minimizing the risk of autoimmunity. Taken together, the results obtained in this study demonstrate that tumors can be efficiently controlled and cured in the evolving field of precision medicine, particularly through the application of personalized immunotherapy.

## Data Availability Statement

The datasets presented in this study can be found in online repositories. The names of the repository/repositories and accession number(s) can be found in the article/**Supplementary Material**.

## Ethics Statement

The studies involving human participants were reviewed and approved by Medical Ethics Committee of Shanghai East Hospital. The patients/participants provided their written informed consent to participate in this study. The animal study was reviewed and approved by Biotherapy Center, Qingdao Central Hospital, The Second Affiliated Hospital, Qingdao University.

## Author Contributions

WZ, QY, and HH designed and performed experiments, analyzed data, and wrote the paper. JL, HQ, WZ, and XS performed experiments. SC translated the paper. WS and YD designed experiments, oversaw the data analysis and wrote the paper. QL conceived the study and wrote the paper. All authors contributed to the article and approved the submitted version.

## Funding

This work was supported by Clinical Plateau Discipline Project in Shanghai Pudong New Area(PWYgy2018-06), Scientific Research Project by Shanghai Science and Technology Committee(19411950400) and Qingdao Outstanding Health Professional Development Fund; Qingdao Science and Technology Project (for Benefiting the People, 19-6-1-27-nsh).

## Conflict of Interest

The authors declare that the research was conducted in the absence of any commercial or financial relationships that could be construed as a potential conflict of interest.

## Supplementary Material

Supplementary Figure 1 Immunogenicity of Cancer-associated mutations in HLA-A2.1/KbTg mice. Splenocytes of mice vaccinated with peptides and polyinosinic: polycytidylic acid (polyI: C) were tested for recognition of mutated peptides by flow cytometry.

Supplementary Figure 2 Expression of HLA-A*0201/Kb chimeric gene in T2/Kb and H522/Kb cells. T2/Kb and H522/Kb cells were stable transfectants and express the product of the HLA-A*0201/Kb chimeric gene (the α1 and α2 domains from HLA-A*0201 and the α3 domain of H-2Kb)

Supplementary Figure 3 Immunogenicity of peptides-containing neoantigen at different various concentrations. Peptide titration of the NRT lines specific for PLACG1-M425L, BCAR1-G23V and ACAD8-T105I peptides. The immune splenocytes from HLA-0201/Kb vaccinated animals were stimulated with various concentrations (100, 10 or 1 μM) of PLACG1-M425L, BCAR1-G23V and ACAD8-T105I peptides. After 4 h of incubation, frequency of IFN-γ-secreting cells was measured. The data are presented as the means ± s.e.m.s from three independent experiments.
