# Supplementary figures and images for: Personal Neoantigens From Patients With NSCLC Induce Efficient Antitumor Responses
Source: Front Oncol. 2021 Apr 13;11:628456. doi: 10.3389/fonc.2021.628456 (PMC8076796; doi:10.3389/fonc.2021.628456)

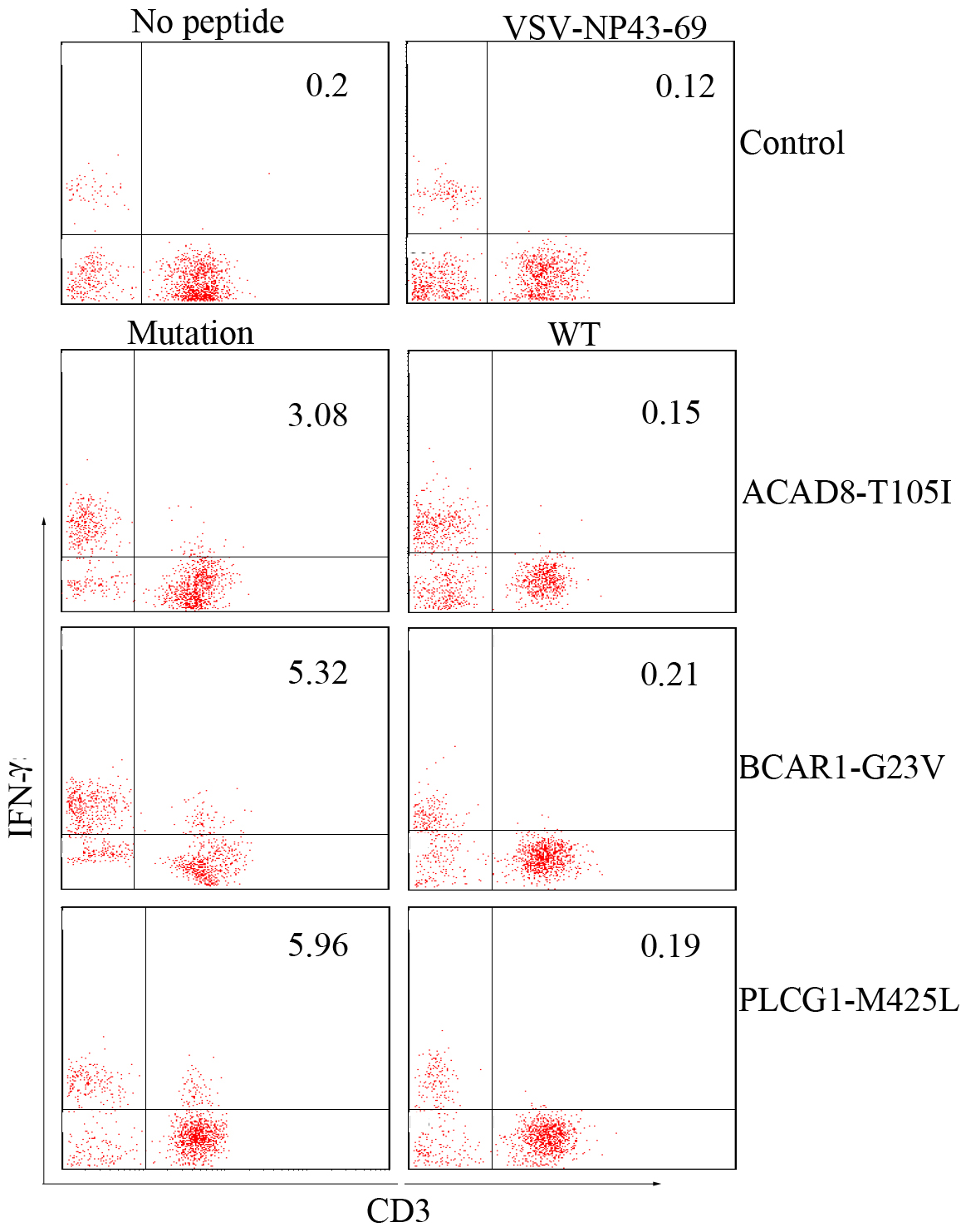

Supplement: Supplementary Figure 1 — Immunogenicity of Cancer-associated mutations in HLA-A2.1/KbTg mice. Splenocytes of mice vaccinated with peptides and polyinosinic: polycytidylic acid (polyI: C) were tested for recognition of mutated peptides by flow cytometry. [file Image_1.tif]

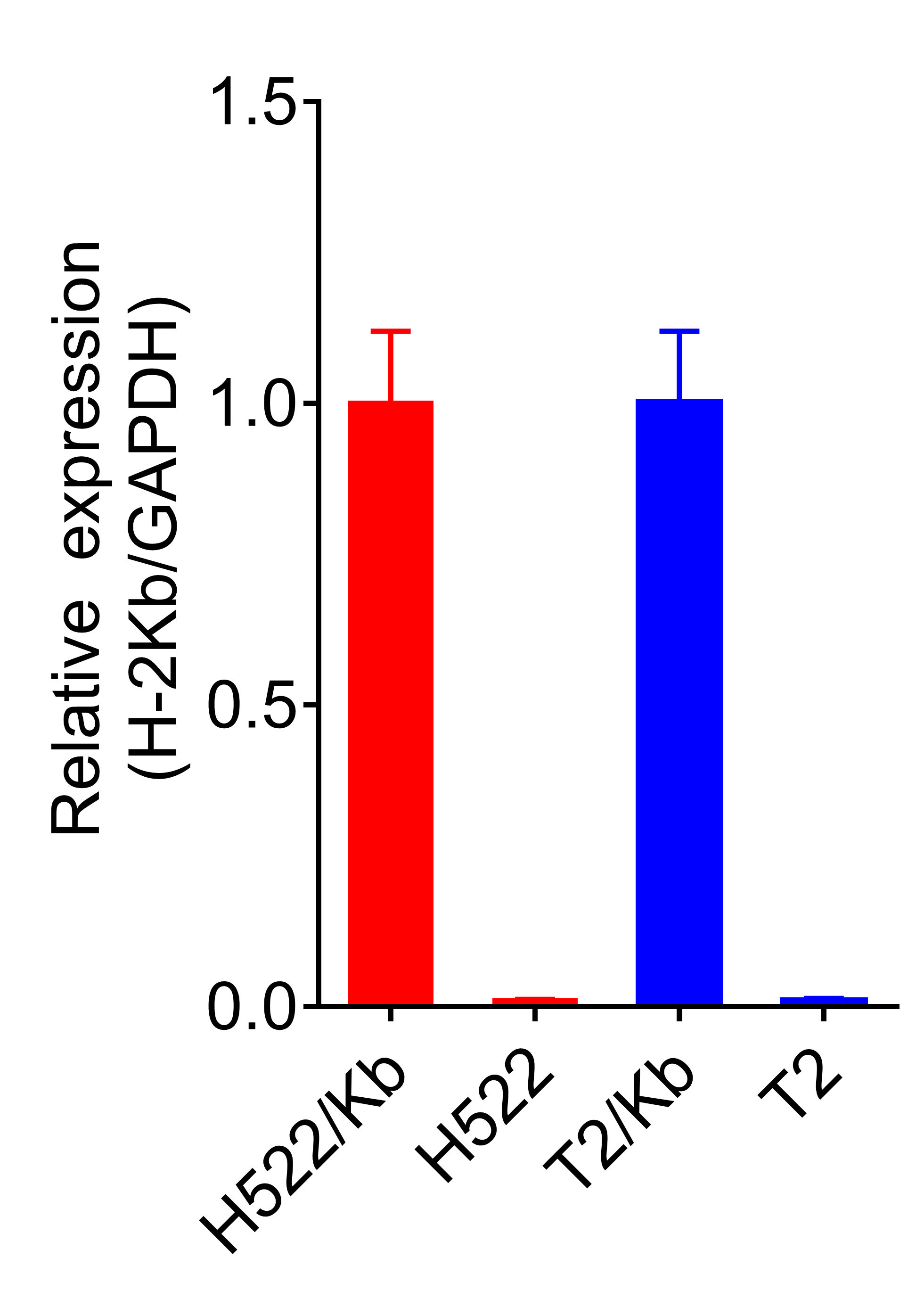

Supplement: Supplementary Figure 2 — Expression of HLA-A*0201/Kb chimeric gene in T2/Kb and H522/Kb cells. T2/Kb and H522/Kb cells were stable transfectants and express the product of the HLA-A*0201/Kb chimeric gene (the α1 and α2 domains from HLA-A*0201 and the α3 domain of H-2Kb) [file Image_2.tif]

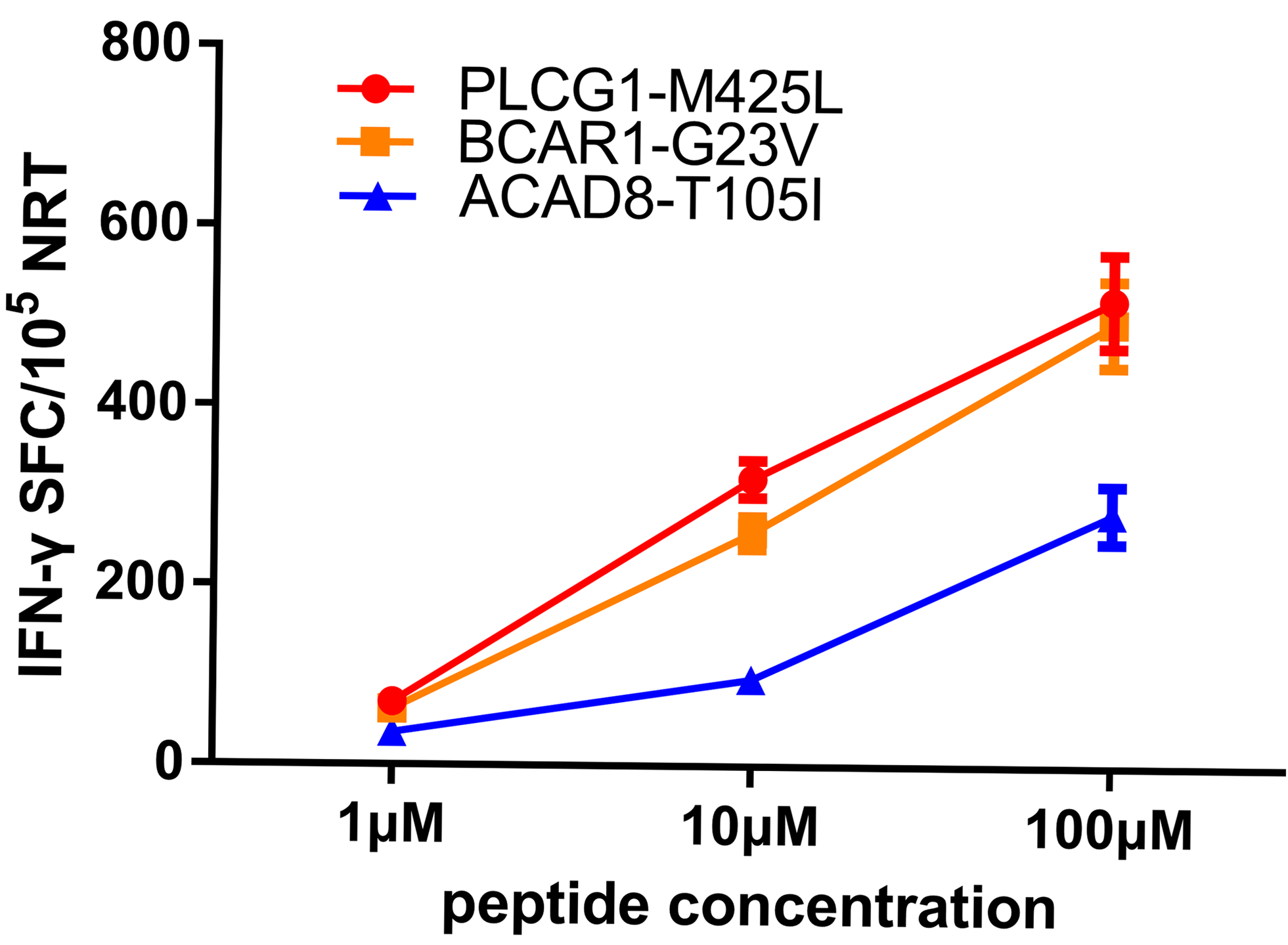

Supplement: Supplementary Figure 3 — Immunogenicity of peptides-containing neoantigen at different various concentrations. Peptide titration of the NRT lines specific for PLACG1-M425L, BCAR1-G23V and ACAD8-T105I peptides. The immune splenocytes from HLA-0201/Kb vaccinated animals were stimulated with various concentrations (100, 10 or 1 μM) of PLACG1-M425L, BCAR1-G23V and ACAD8-T105I peptides. After 4 h of incubation, frequency of IFN-γ-secreting cells was measured. The data are presented as the means ± s.e.m.s from three independent experiments. [file Image_3.tif]
