# Supplementary material for: Personal Neoantigens From Patients With NSCLC Induce Efficient Antitumor Responses
Source: Front Oncol. 2021 Apr 13;11:628456. doi: 10.3389/fonc.2021.628456 (PMC8076796; doi:10.3389/fonc.2021.628456)
Supplement: Supplementary file 4 [file DataSheet_1.zip › Supplementary Table 1.DOCX]

**Supplementary Table 1.** HLA alleles of 3 patients with NSCLC

| Patient ID | HLA I | | | HLA II | | | | | |
| --- | --- | --- | --- | --- | --- | --- | --- | --- | --- |
|  | A | B | C | DQA1 | DQB1 | DRB1 | DRA | DPA1 | DPB1 |
| P01 | A*24:02 A*02:06 | B*15:02 B*81:01 | C*08:01 C*08:01 | DQA1*04:01 DQA1*05:01 | DQB1*03:01 DQB1*03:01 | DRB1*11:01 DRB1*12:02 | DRA*01:01 DRA*01:01 | DPA1*01:03 DPA1*01:03 | DPB1*21:01 DPB1*05:01 |
| P02 | A*33:03 A*02:07 | B*58:01 B*15:01 | C*03:04 C*03:04 | DQA1*03:01 DQA1*05:01 | DQB1*03:03 DQB1*02:02 | DRB1*03:01 DRB1*09:01 | DRA*01:01 DRA*01:01 | DPA1*02:02 DPA1*02:02 | DPB1*105:01 DPB1*05:01 |
| P03 | A*11:01 A*02:01 | B*40:01 B*51:01 | C*14:02 C*07:02 | DQA1*01:02 DQA1*01:02 | DQB1*06:02 DQB1*06:02 | DRB1*15:01 DRB1*16:08 | DRA*01:01 DRA*01:01 | DPA1*01:03 DPA1*01:03 | DPB1*02:01 DPB1*02:01 |
