# Supplementary material for: Personal Neoantigens From Patients With NSCLC Induce Efficient Antitumor Responses
Source: Front Oncol. 2021 Apr 13;11:628456. doi: 10.3389/fonc.2021.628456 (PMC8076796; doi:10.3389/fonc.2021.628456)
Supplement: Supplementary file 4 [file DataSheet_1.zip › Supplementary Table 3.DOCX]

Supplementary Table 3. Clinicopathological characteristics of 3 NSCLC patients

| Patient | Sex | Age | Smoking status (py) | Stage | TNM | Anatomic site | Pathological type | First-line treatment | Prognosis |
| --- | --- | --- | --- | --- | --- | --- | --- | --- | --- |
| P01 | Male | 57 | 0-10 | IV | T2N3M1 | Left lung | SC | Chemoradiotherapy(GC) | PD (dead) |
| P02 | Male | 67 | 0-10 | IV | T4N3M1 | Right lung | SC | Chemotherapy(GP) | PR |
| P03 | Male | 78 | 0-10 | IIIB | T4N0M0 | Right lung | SC | - Surgery, Chemotherapy(GC) | SD |

Py: packs/year

SC: Squamous carcinoma

PD: Progressive disease

SD: Stable disease

PR: Partial response

GC; G: Gemcitabine, C: carboplatin

GP; G: Gemcitabine, P: cis-platinum
